# Supplementary material for: Autodesmotic reactions for general strain energy evaluation in polycyclic aromatic nanocarbons
Source: Commun Chem. 2025 Dec 16;9:41. doi: 10.1038/s42004-025-01848-w (PMC12824410; doi:10.1038/s42004-025-01848-w)
Supplement: Supplementary file 2 — Supplementary Information [file 42004_2025_1848_MOESM2_ESM.pdf]

Supplementary Information

Autodesmotic reactions for general strain energy evaluation in polycyclic aromatic nanocarbons

Yang Wang*,†,‡

†*School of Chemistry and Chemical Engineering, Yangzhou University, Yangzhou, Jiangsu 225002, China*

‡*Jiangsu Provincial Key Laboratory of Green and Functional Materials and Environmental Chemistry, Yangzhou University, Yangzhou, Jiangsu 225002, China*

E-mail: yangwang@yzu.edu.cn

Contents

Note 1: Unbalanced π-Energy in Hyperhomodesmotic Reactions **3**

Note 2: Comparing SE predictions between Autodesmotic and StrainViz methods **4**

Note 3: Regression Analysis of Total Energies for (n, n) CNBs **6**

Note 4: Balance of π-Energy in Homodesmotic Reactions for SE evaluation of Septulene and Corannulene **7**

Note 5: Enumeration and Selection of $C_{48}H_{24}$ PAH isomers **8**

Proof That a $C_{48}H_{24}$ Benzenoid PAH Contains Exactly 13 Rings 8

Enumeration and Selection of $C_{48}H_{24}$ Reference Molecules 10

Note 6: Distribution of C−H Bond Lengths and H···H Distances **11**

Note 7: Derivation of Eq. 5 in the Main Text **13**

Note 8: Model Performance for $C_{48}H_{24}$ PAHs **15**

Note 9: Optimized Model Parameters **16**

Energy Model for $C_{48}H_{24}$ PAH isomers 16

Bond Length Model for $C_{48}H_{24}$ PAH isomers 16

General Energy Model for PAHs from C_6H_6 to $C_{96}H_{24}$ 19

Bond Length Model for PAHs from C_6H_6 to $C_{96}H_{24}$ 19

Note 10: Bond Types for Predicting Bond Lengths **22**

Note 11: Validation of Bond Length Models on Benchmark CNBs **25**

Note 12: Performance of Models Employing the Simple and Distance-Dependent HMO Methods **26**

Note 13: Validation of Ground-State Diagnosis by UBS-DFT **29**

Supplementary References **31**

Note 1: Unbalanced π-Energy in Hyperhomodesmotic Reactions

We present a straightforward example demonstrating that a hyperhomodesmotic reaction does not necessarily ensure the balance of π-energy.

As shown in Fig. S1, the two selected planar benzenoid isomers of $C_{48}H_{24}$ can be interconverted via a hyperhomodesmotic reaction, as both compounds contain equal numbers of carbon–carbon bond types (with 8 HC=CH, 2 HC−CH, 8 HC=C, 20 HC−C, 8 C=C, and 14 C−C bonds) and all carbon atoms are of sp^2 type. However, the isomer in Fig. S1a has a π-energy 15.5 kcal/mol lower than that of the isomer in Fig. S1b, as evaluated from the energy prediction model for planar $C_{48}H_{24}$ benzenoid PAHs (see the Results section in the main text).

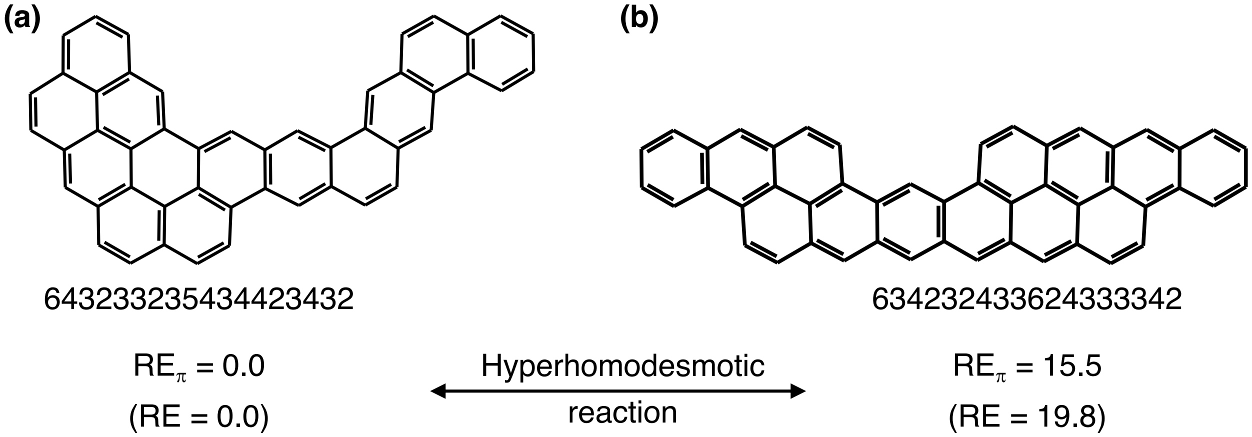

Figure S1. Two distinct planar benzenoid isomers of $C_{48}H_{24}$ with boundary-edges (BEC) codes[1,2] of (a) 643233235434423432 and (b) 634232433624333342. The interconversion between them corresponds to a hyperhomodesmotic reaction, preserving both the numbers of carbon–carbon bond types and the numbers of carbon atom hybridization types. The relative DFT energies (RE) and relative π-energies (RE_π) of both compounds are given in kcal/mol. The π-energies were evaluated using the predictive model for planar $C_{48}H_{24}$ benzenoid PAHs.

Furthermore, an even larger difference is observed in the their total DFT energies (19.8 kcal/mol), as shown in Fig. S1, indicating that different choices of reference compounds in hyperhomodesmotic reactions may lead to significant errors in strain energy evaluation.

Note 2: Comparing SE predictions between Autodesmotic and StrainViz methods

For many $C_{48}H_{24}$ CNBs, the asymptotic model is inapplicable due to significant deviations of their total energies from the required parabolic size dependence. Therefore, in the following assessment, we compare the autodesmotic model results with those from StrainViz for 22 selected $C_{48}H_{24}$ CNBs. These CNBs represent a variety of structural types, including tubular, conical, quasiplanar, and Möbius forms, with representative examples illustrated in Fig. S2a.

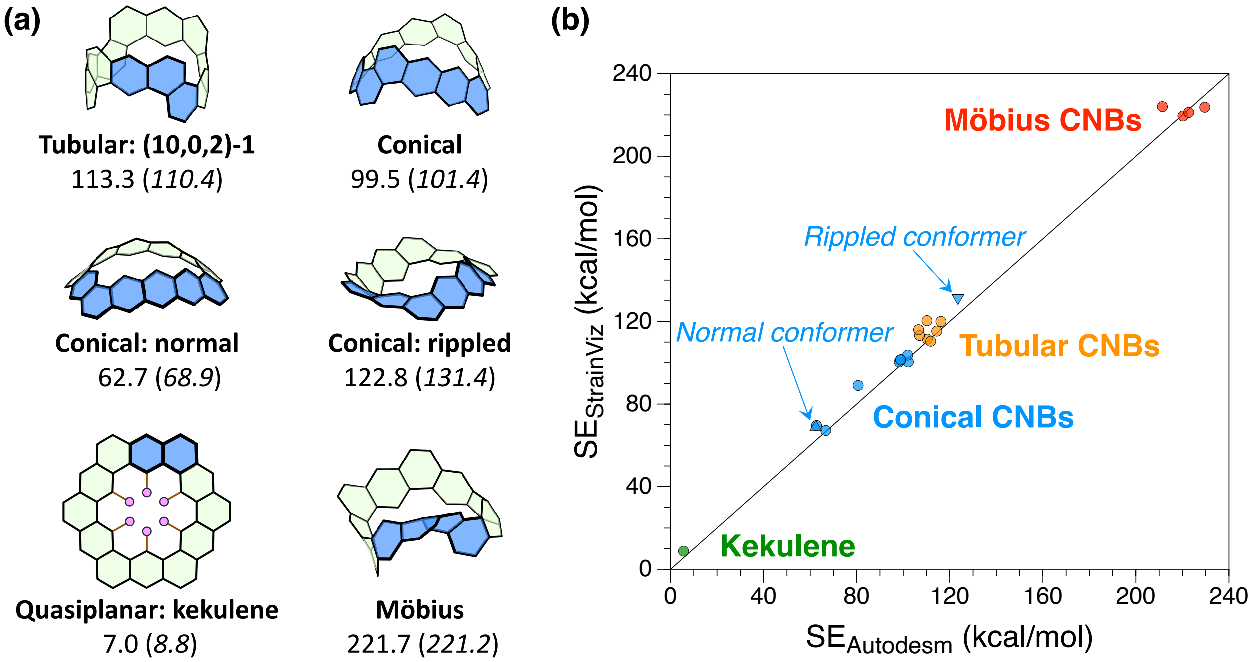

Figure S2. (a) Representative $C_{48}H_{24}$ CNBs of distinct structural types used for evaluation. The repeat unit is highlighted in blue. The internal hydrogen atoms in kekulene are marked with pink circles. SEs (in kcal/mol) predicted by the autodesmotic and StrainViz[3] models are shown outside and inside parentheses, respectively. (b) Comparison of SEs obtained from the autodesmotic and StrainViz[3] models for 22 $C_{48}H_{24}$ CNBs. The two distinct conformers of the same conical CNB are indicated by blue triangles.

As shown in Fig. S2b, the autodesmotic and StrainViz models yield consistent SE predictions for these structurally diverse CNBs. The evaluated SEs exhibit chemically intuitive trends, detailed as follows. Möbius CNBs (red points in Fig. S2b) possess the highest strain energies, exceeding 200 kcal/mol, as a consequence of the substantial twist of the entire molecular belt. At the opposite extreme, the kekulene molecule[4–8] (green point) shows an SE of only a few kcal/mol, primarily attributed to a slight distortion from the planar D_{6h}

geometry to a nonplanar D_{3d} configuration induced by steric hindrance among six internal hydrogen atoms (highlighted in pink in Fig. S2a).[8]

Tubular and conical CNBs display intermediate SE values, represented by the orange and blue points in Fig. S2b, respectively. A tubular CNB can be viewed as a molecular segment excised from the sidewall of a corresponding CNT.[9–13] Owing to its cylindrical carbon framework, all benzenoid rings are aligned parallel to the tubular axis, with their ring normals along the radial direction of the macrocycle. Consequently, efficient overlap occurs predominantly between the inner lobes of the carbon π orbitals, while overlap between the outer lobes is substantially reduced. This compromised π conjugation largely accounts for the relatively high SEs observed in tubular CNBs. By contrast, conical CNBs can be regarded as intermediates between tubular and planar forms, where the π orbital lobes on both sides of the benzenoid rings achieve more balanced overlap, resulting in overall more effective π conjugation. Thus, conical CNBs generally exhibit lower SEs than tubular CNBs.

Additionally, both the autodesmotic and StrainViz models effectively capture the pronounced difference in SE between two conformations of a conical CNB. As shown in the middle row of Fig. S2a, the rippled conformer has nearly twice the SE of the normal conformer: 122.8 vs. 62.7 kcal/mol according to the autodesmotic model, and 131.4 vs. 68.9 kcal/mol according to StrainViz.

Note 3: Regression Analysis of Total Energies for (n, n) CNBs

We applied the asymptotic model[14] to a benchmark series of armchair (n, n) CNBs[15–18] with $n = 2, 4, \ldots, 30$ (see Fig. 4 in the main text for structures with $n = 6, 8$, and 12). As shown by the black linear regression line in Fig. S3, the DFT-computed total energy per n correlates excellently with $1/n^2$ for n ranging from 4 to 16, yielding $R^2 = 0.999994$ and an RMSE of 0.46 kcal/mol. In contrast, the smallest system, [4]CNB ($n = 2$; blue triangle in Fig. S3), displays a significant deviation from the linear regression, differing by 38.95 kcal/mol. The departure of [4]CNB (2,2) from the quadratic energy–size relationship indicates that the approximations in the continuum elasticity model for curved carbon shells[19,20] are no longer valid for very small CNBs.

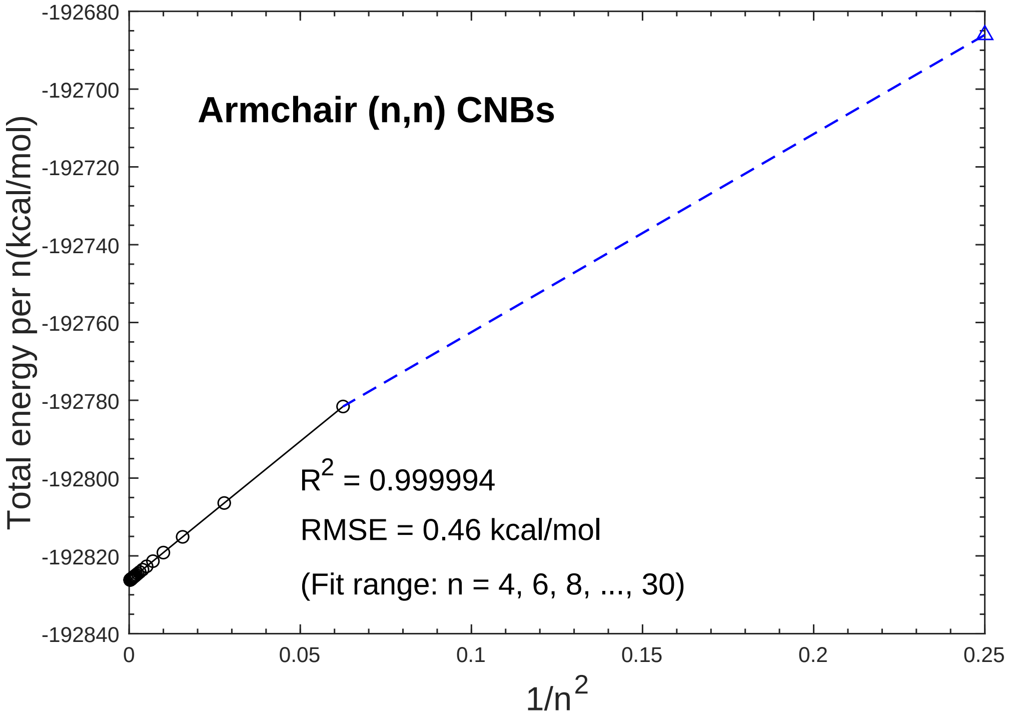

Figure S3. DFT total energy as a function of n for armchair (n, n) CNBs[15–18] with $n = 2, 4, 6, \ldots, 30$. Black circles denote CNBs with $n = 4, 6, \ldots, 30$, for which the linear regression is shown as a black line. The smallest [2]CNB ($n = 2$) is indicated by a blue triangle. A blue dashed line highlights the deviation of the $n = 2$ case from the linear regression fitted to $n \geqslant 4$.

Note 4: Balance of π-Energy in Homodesmotic Reactions for SE evaluation of Septulene and Corannulene

In the SE evaluation of septulene,[21] the following homodesmotic reaction was used:

$$C_{56}H_{28}(\text{septulene}) \longrightarrow \frac{7}{6}C_{48}H_{24}(\text{kekulene}) + \Delta E_{\pi} \tag{1}$$

where $\Delta E_{\pi} = \frac{7}{6}E_{\pi}(\text{kekulene}) - E_{\pi}(\text{septulene})$ represents the difference in π-energy for this reaction. The π-energy of each species was estimated using our general energy prediction model trained on benzenoid PAHs ranging from C_6H_6 to $C_{96}H_{48}$ (see the Results section in the main text). This yields $\Delta E_{\pi} = -0.2$ kcal/mol for reaction 1, indicating that the π-energies of kekulene and septulene are nearly identical.

The homodesmotic reaction used for SE evaluation of corannulene[22] was

$$C_{20}H_{10}(\text{corannulene}) \longrightarrow \frac{5}{6}C_{24}H_{12}(\text{coronene}) + \Delta E_{\pi} \tag{2}$$

with $\Delta E_{\pi} = \frac{5}{6}E_{\pi}(\text{coronene}) - E_{\pi}(\text{corannulene})$. Using our general energy prediction model, we obtain $\Delta E_{\pi} = -4.1$ kcal/mol, suggesting the approximate balance of π-energy in homodesmotic reaction 2.

Note 5: Enumeration and Selection of $C_{48}H_{24}$ PAH isomers

Proof That a $C_{48}H_{24}$ Benzenoid PAH Contains Exactly 13 Rings

We first demonstrate that all possible benzenoid isomers of $C_{48}H_{24}$ contain exactly 13 rings. A benzenoid PAH structure can be represented as a polyhex graph consisting of H hexagonal faces and E edges. The edges fall into two categories: those shared between adjacent hexagons and those forming the perimeter of the structure (i.e., boundary edges, each belonging to only one hexagon). Let S and B denote the numbers of shared and boundary edges, respectively.

Each hexagon has six edges, so the total number of edge occurrences (with shared edges counted twice) is $6H$. Each shared edge contributes to two hexagons and is thus counted twice, while each boundary edge is counted once. Therefore, the number of boundary edges is

$$B = 6H - 2S \tag{3}$$

The total number of edges in the polyhex graph is then

$$E = S + B = 6H - S \tag{4}$$

According to Euler's formula for planar graphs,

$$V - E + H = 1 \tag{5}$$

where V is the total number of vertices (i.e., carbon atoms). Substituting Eq. (4) into Eq. (5) yields

$$V = 5H - S + 1 \tag{6}$$

In a polyhex graph, vertices can be classified according to the number of hexagons they belong to (see Fig. S4 for an example):

- V_1: vertices belonging to only one hexagon
- V_2: vertices shared by two hexagons
- V_3: vertices shared by three hexagons

Thus,

$$V = V_1 + V_2 + V_3 \tag{7}$$

$$6H = V_1 + 2V_2 + 3V_3 \tag{8}$$

The left-hand side of Eq. (8) counts all vertex occurrences across all hexagons, allowing shared vertices to be counted multiple times, while the right-hand side properly accounts for the multiplicity of each vertex type.

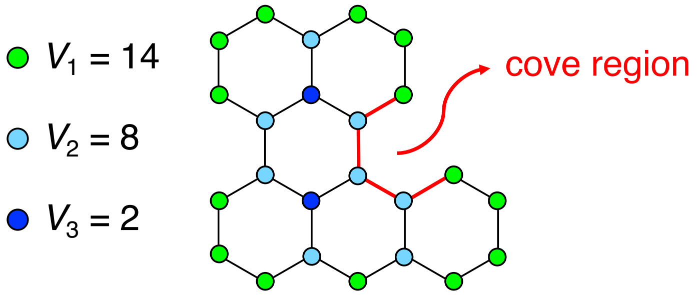

Figure S4. Graph of benzo[a]perylene ($C_{24}H_{14}$). Three types of vertices are distinguished by color, with V_1, V_2, and V_3 being their counts. The cove region[23,24] is highlighted in red.

Alternatively, the number of vertices can be related to the number of hexagons and shared edges as

$$V = 6H - 2S + V_3 \tag{9}$$

Here, the first term ($6H$) counts the total vertex occurrences, including overcounting; the second term ($-2S$) removes double-counting introduced by the S shared edges (each contributing two extra appearances); the final term ($+V_3$) corrects for over-subtraction by the second term: each vertex shared by three hexagons is subtracted three times but should only be subtracted twice, so one occurrence is restored.

Subtracting Eq. (9) from Eq. (8) and noticing that $V = V_1 + V_2 + V_3$ (Eq. (7)), we obtain

$$V_1 = 3V + 2S - 12H \tag{10}$$

Combining Eqs. (10) and (6) to eliminate S, we get

$$H = \frac{V - V_1}{2} + 1 \tag{11}$$

For a benzenoid molecule C_nH_m, it is evident that $V = n$ and $V_1 = m$ (since V_1 corresponds to carbon atoms of CH type, whose number is exactly the number of hydrogen atoms). Substituting into Eq. (11), the number of rings is

$$H = \frac{n - m}{2} + 1 \tag{12}$$

Therefore, in the specific case of $C_{48}H_{24}$, each benzenoid PAH molecule contains exactly

$(48-24)/2+1=13$ rings.

Enumeration and Selection of $C_{48}H_{24}$ Reference Molecules

Given that planar benzenoid PAHs with the formula $C_{48}H_{24}$ contain exactly 13 rings, we conducted a complete enumeration of planar polyhex graphs with 13 hexagons using the boundary-edges code (BEC) algorithm.[1,2] This yielded a total of 3,198,256 distinct graphs, in exact agreement with previous work.[25] Among these, 114,326 graphs satisfy the conditions $V=48$ and $V_1=24$ (as verified using Eqs. (6) and (10)), corresponding to $C_{48}H_{24}$ benzenoid isomers.

Applying the simple Hückel molecular orbital (HMO) theory,[26–29] we further excluded all potentially open-shell structures with a HOMO–LUMO gap smaller than 0.001 $|\beta|$ (β being resonance integral), resulting in 83,475 candidate isomers. We then screened out all structures containing cove regions,[23,24] defined as concave edge motifs formed by four fused benzene rings in a U-shaped arrangement (highlighted by the red lines in Fig. S4), which inevitably lead to nonplanar geometries due to steric repulsion between close H···H contacts. This screening left 7,675 remaining candidate isomers.

To identify the valid $C_{48}H_{24}$ structures for use as reference molecules in strain energy evaluation, we applied two additional criteria: (1) a planar equilibrium geometry and (2) a closed-shell ground state. Accordingly, we carried out full geometry optimizations and vibrational frequency analyses for all 7,675 candidate isomers at the GFN2-xTB[30,31] level using the XTB 6.3.3 package. Excluding all isomers with nonplanar equilibrium structures reduced the set to 2,427 candidates, for which we performed single-point B3LYP/3-21G calculations. Unrestricted broken spin-symmetry DFT[32] calculations revealed 767 of these isomers have an open-shell singlet ground state, and were therefore excluded. The remaining 1,660 isomers were fully optimized at the B3LYP/6-31G* level, and harmonic vibrational frequency analyses confirmed that all are true minima on the potential energy surface. After further discarding nonplanar structures, we obtained 1,516 planar benzenoid isomers of $C_{48}H_{24}$. Finally, single-point B3LYP/6-311G* calculations verified that all these reference molecules possess closed-shell ground states.

Note 6: Distribution of C−H Bond Lengths and H···H Distances

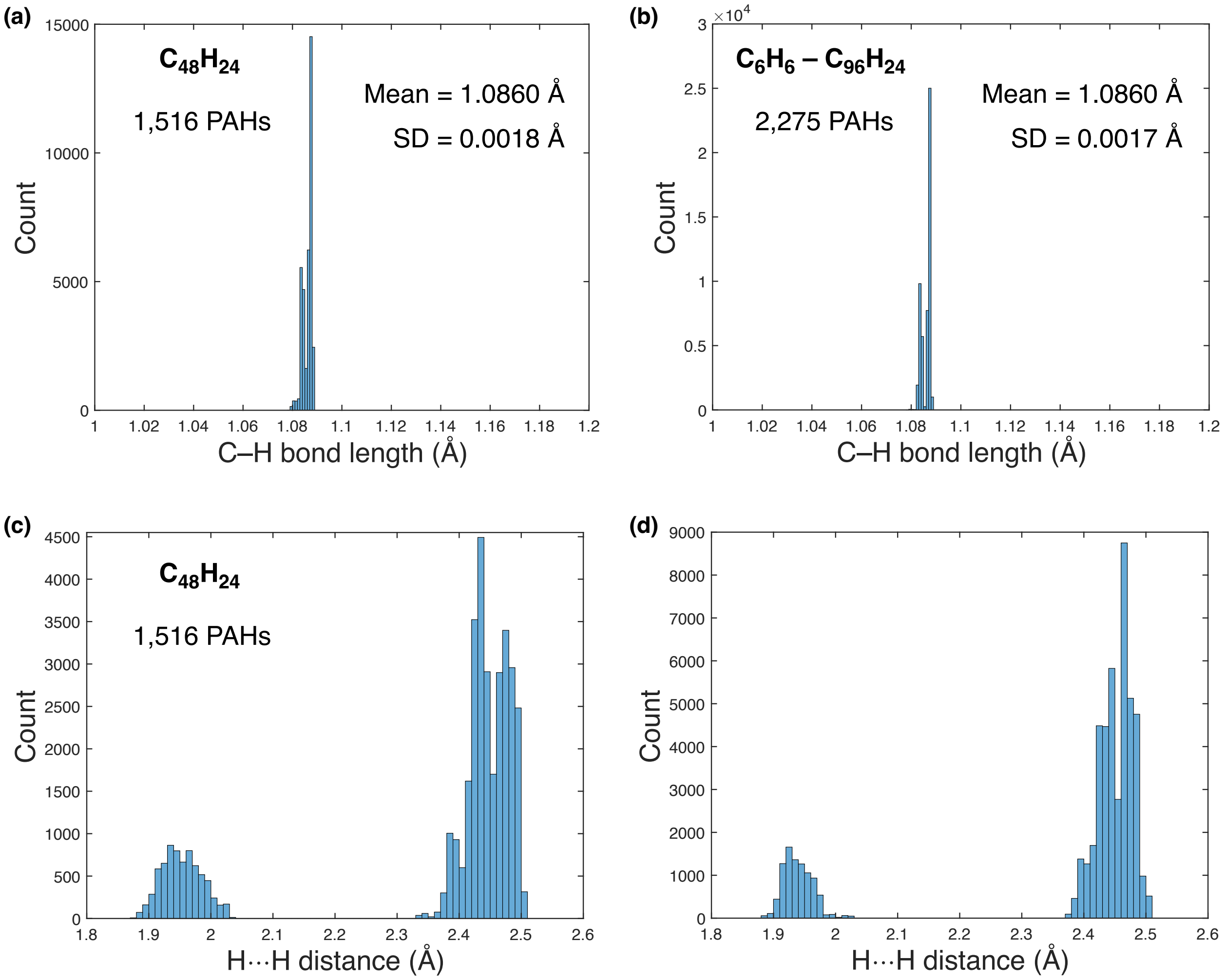

Figure S5. Interatomic distance distributions for all reference PAH molecules in the datasets. (a) C−H bond lengths in $C_{48}H_{24}$ isomers. (b) C−H bond lengths in PAHs ranging from C_6H_6 to $C_{96}H_{24}$. (c) H···H distances in $C_{48}H_{24}$ isomers. (d) H···H distances in PAHs from C_6H_6 to $C_{96}H_{24}$. H···H distances longer than 3.0 Å are excluded.

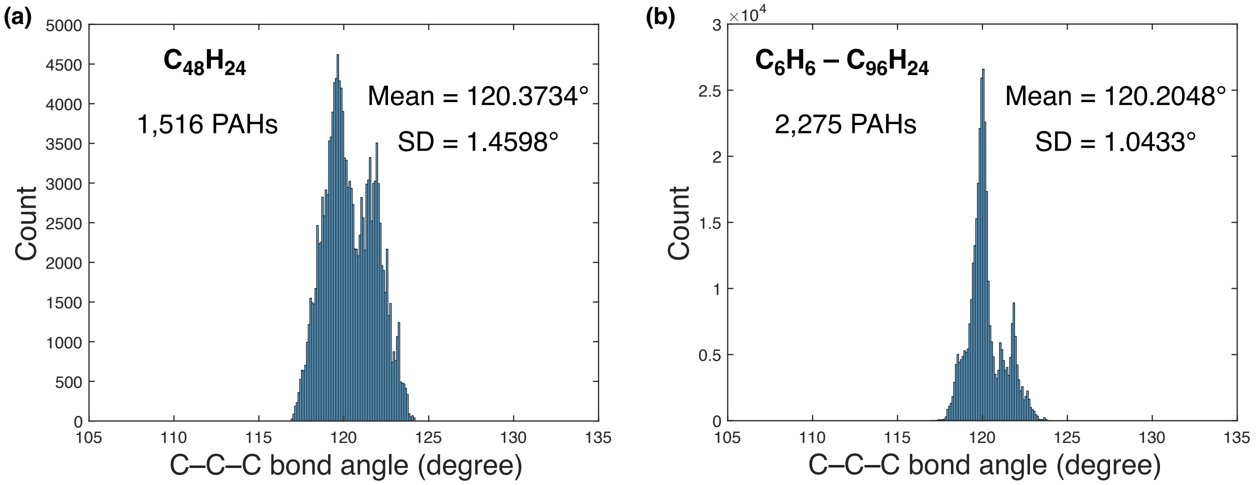

Figure S6. C−C−C bond angle distributions for all reference PAH molecules in both datasets: (a) $C_{48}H_{24}$ isomers. (b) PAHs ranging from C_6H_6 to $C_{96}H_{24}$.

Note 7: Derivation of Eq. 5 in the Main Text

We reproduce Eq. 5 from the main text below:

$$\begin{aligned}\Delta E_{\text{ref}} = \Big(E_\pi + E_{\text{C–C}} + E_{\text{H}\cdots\text{H}} + E_0\Big) - \frac{4m-n}{18}\Big(E^*_\pi + E^*_{\text{C–C}} + E^*_0\Big) \\ - \frac{n-m}{72}\Big(E^{**}_\pi + E^{**}_{\text{C–C}} + E^{**}_0\Big)\end{aligned} \tag{13}$$

where superscripts * and ** denote quantities corresponding to benzene and circumcircumcoronene, respectively.

Since the total number of C–H bonds (equal to the total number of H atoms) and the total number of core electrons are balanced in the reference reaction (Eq. 3 in the main text), it is reasonable to assume that the constant terms in Eq. 1 of the main text for each species (namely, E_0, E^*_0, and E^{**}_0 for C_nH_m, benzene, and circumcircumcoronene, respectively), largely cancel out, leaving an approximately size-independent residual constant. That is,

$$E_0 - \frac{4m-n}{18}E^*_0 - \frac{n-m}{72}E^{**}_0 = \epsilon. \tag{14}$$

Hence,

$$\begin{aligned}\Delta E_{\text{ref}} &= E_\pi + E_{\text{C–C}} + E_{\text{H}\cdots\text{H}} - \frac{4m-n}{18}\Big(E^*_\pi + E^*_{\text{C–C}}\Big) \\ &\quad - \frac{n-m}{72}\Big(E^{**}_\pi + E^{**}_{\text{C–C}}\Big) + \epsilon \\ &= E_\pi + E_{\text{C–C}} + E_{\text{H}\cdots\text{H}} + \left(\frac{E^*_\pi + E^*_{\text{C–C}}}{18} - \frac{E^{**}_\pi + E^{**}_{\text{C–C}}}{72}\right)n \\ &\quad + \left[\frac{E^{**}_\pi + E^{**}_{\text{C–C}}}{72} - \frac{2(E^*_\pi + E^*_{\text{C–C}})}{9}\right]m + \epsilon\end{aligned} \tag{15}$$

Applying the expressions for E_π, $E_{\text{C–C}}$, and $E_{\text{H}\cdots\text{H}}$ from the main text (see Eq. 2), Eq. (15) becomes

$$\begin{aligned}\Delta E_{\text{ref}} = 2\beta\sum_{i=1}^{n/2}\chi_i + \gamma\sum_{i}^{\text{C–C}}(d_i/d_0 - 1)^\lambda + \eta\, n_{\text{H}\cdots\text{H}} + \left(\frac{E^*_\pi + E^*_{\text{C–C}}}{18} - \frac{E^{**}_\pi + E^{**}_{\text{C–C}}}{72} + \alpha\right)n \\ + \left[\frac{E^{**}_\pi + E^{**}_{\text{C–C}}}{72} - \frac{2(E^*_\pi + E^*_{\text{C–C}})}{9}\right]m + \epsilon\end{aligned} \tag{16}$$

Defining

$$\nu = \frac{E_{\pi}^{*} + E_{\mathrm{C-C}}^{*}}{18} - \frac{E_{\pi}^{**} + E_{\mathrm{C-C}}^{**}}{72} + \alpha, \tag{17}$$

and

$$\mu = \frac{E_{\pi}^{**} + E_{\mathrm{C-C}}^{**}}{72} - \frac{2(E_{\pi}^{*} + E_{\mathrm{C-C}}^{*})}{9}, \tag{18}$$

we arrive at the final form:

$$\Delta E_{\mathrm{ref}} = 2\beta \sum_{i=1}^{n/2} \chi_i + \gamma \sum_{i}^{\mathrm{C-C}} (d_i/d_0 - 1)^{\lambda} + \eta\, n_{\mathrm{H\cdots H}} + \nu\, n + \mu\, m + \epsilon. \tag{19}$$

Note 8: Model Performance for $C_{48}H_{24}$ PAHs

As shown in Fig. S7a, the model accurately predicts the total DFT energies of all 1,516 $C_{48}H_{24}$ PAH isomers, with a squared correlation coefficient (R^2) of 0.9936 and a root-mean-square error (RMSE) of less than 0.4 kcal/mol. Fig. S7b shows strong performance of the bond length model in predicting DFT-optimized C−C bond lengths for all 1,516 reference $C_{48}H_{24}$ PAHs ($R^2 > 0.995$, RMSE < 0.002 Å).

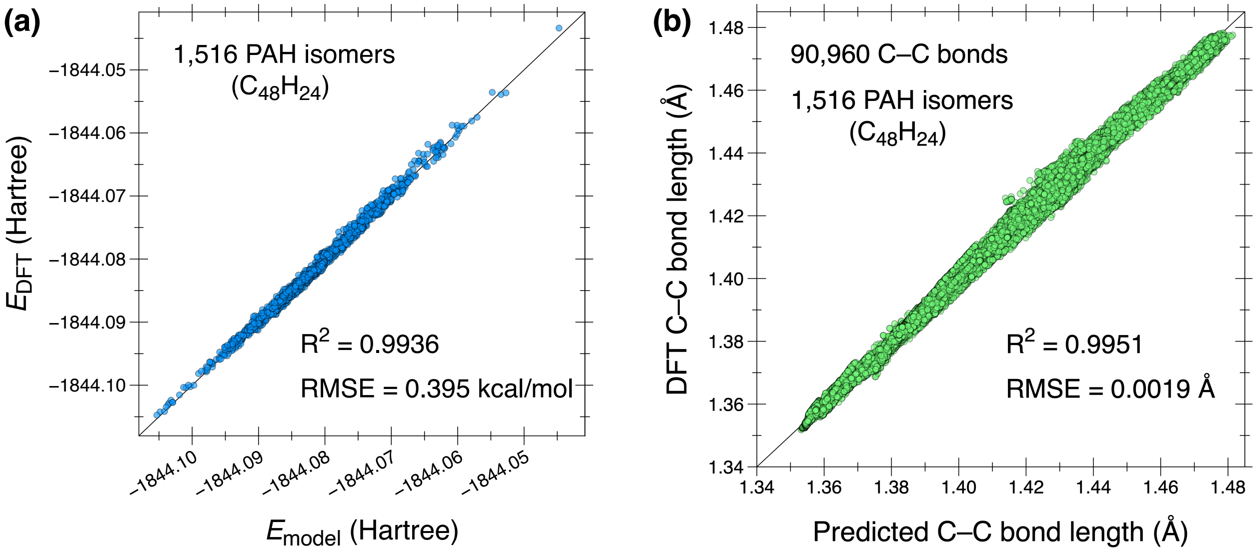

Figure S7. Comparison of (a) total energies and (b) C−C bond lengths obtained from DFT calculations and model predictions (Eqs. 2 and 7 in the main text, respectively) for 1,516 planar benzenoid PAHs, comprising a total of 90,960 C–C bonds. R^2 and RMSE are indicated in each panel.

Note 9: Optimized Model Parameters

Energy Model for $C_{48}H_{24}$ PAH isomers

By performing least-squares fit to the DFT total energies (E_{tot}, in Hartree) of 1,516 reference molecules of $C_{48}H_{24}$ PAHs, we obtained the following optimized parameters for the model (Eq. 2 in the main text):

- $\beta = -0.0803138173080486$ Hartree
- $\gamma = 0.278172493862349$ Hartree, $\lambda = 0.4331061178$, $d_0 = 1.2951279916$ Å
- $\eta = 0.00142502255575605$ Hartree
- $\kappa = -1844.50138198843$ Hartree

Notably, the fitted value of the resonance integral, $\beta = -2.19$ eV, is consistent with the value of -2.49 eV reported in earlier studies.[33,34] The fitted average H$\cdots$H repulsion energy, $\eta = 0.89$ kcal/mol, aligns well with known energetic trends and chemical intuition.[35,36] For example, phenanthrene, which contains a single close H$\cdots$H contact, is 4.2 kcal/mol more stable than anthracene, which has none.[35,36] Upon converting both to their 4,5-didehydro derivatives, which removes the H$\cdots$H contact, 4,5-didehydrophenanthrene becomes 5.2 kcal/mol more stable than 4,5-didehydroanthracene.[35,36] This observation implies that the H$\cdots$H repulsion in phenanthrene contributes roughly 1 kcal/mol of destabilization, in reasonable agreement with our fitted value of η.

Bond Length Model for $C_{48}H_{24}$ PAH isomers

Using a range of values for the hyperparameter ζ in the distance-dependent HMO theory, we performed least-squares fits to the DFT-optimized C−C bond lengths (d_i, in Å) in 1,516 reference molecules of $C_{48}H_{24}$ PAHs. The optimal value of ζ was identified as the one that minimizes the RMSE, and the corresponding optimized model parameters (see Eq. 7 in the main text) are listed below and in Tables S1 and S2.

- $\zeta = 2.70$
- $\rho = 0.148137756599480$

Table S1. Optimized values (in Å) of parameters associated with different atom-based types of C−C bonds in the bond length model for $C_{48}H_{24}$ PAH isomers. See Section for definitions of atom-based bond types.

Parameter	Bond type	Value	Parameter	Bond type	Value
w_1	Type 1	1.332208922130160	w_8	Type 8	1.336839517306866
w_2	Type 2	1.330563662692855	w_9	Type 9	1.340696793227426
w_3	Type 3	1.330996221227133	w_{10}	Type 10	1.331763086411673
w_4	Type 4	1.327862258414070	w_{11}	Type 11	1.341587895270984
w_5	Type 5	1.335596424431933	w_{12}	Type 12	1.341644393415186
w_6	Type 6	1.330599847432850	w_{13}	Type 13	1.338769484043799
w_7	Type 7	1.335924634074002	w_{14}	Type 14	1.335390507218745

Table S2. Optimized values (in Å) of parameters associated with different ring-based types of C−C bonds in the bond length model for $C_{48}H_{24}$ PAH isomers. See Section for definitions of ring-based bond types.

Parameter	Bond type	Value	Parameter	Bond type	Value
u_1	1A	0.001581690634738	u_{25}	2B/2C	−0.002995549143613
u_2	2A	0.000568469645274	u_{26}	2B/3B	−0.000263607741078
u_3	2B	−0.004900478959216	u_{27}	2C/2C	−0.004774923269219
u_4	2C	−0.002639520053261	u_{28}	2C/3B	−0.005420125432773
u_5	3A	−0.004117268262726	u_{29}	2C/3C	−0.004125034415421
u_6	3B	−0.006266561409960	u_{30}	2C/4B	−0.002949715919132
u_7	3C	−0.012229442115753	u_{31}	3A/3A	0.002305578761833
u_8	4A	−0.005167175620422	u_{32}	3A/3B	−0.000523526424281
u_9	4B	−0.012905924086209	u_{33}	3A/4A	−0.001170840880369
u_{10}	4C	−0.009830970498260	u_{34}	3A/4B	−0.001299315232777
u_{11}	5A	−0.011916366575362	u_{35}	3A/4C	−0.001514585820361
u_{12}	1A/2B	0.000000000000000	u_{36}	3A/5A	−0.001893697614190
u_{13}	1A/2C	0.000000000000000	u_{37}	3A/6A	−0.001589016575325
u_{14}	1A/3B	−0.001332782215604	u_{38}	3B/3B	−0.001208403171334
u_{15}	1A/3C	0.000000000000000	u_{39}	3B/4A	−0.001924899018512
u_{16}	1A/4B	−0.001142921716205	u_{40}	3B/4B	−0.004635738384436
u_{17}	2A/2A	0.011442803419505	u_{41}	3B/5A	−0.004748122045493
u_{18}	2A/3A	0.004517784743381	u_{42}	4A/4A	−0.003188935307116
u_{19}	2A/3B	0.003460358686730	u_{43}	4A/4B	−0.003385595684461
u_{20}	2A/4A	0.003834118382835	u_{44}	4A/4C	−0.004044343443834
u_{21}	2A/4B	−0.001663184236109	u_{45}	4A/5A	−0.003070482422433
u_{22}	2A/4C	−0.001516066342591	u_{46}	4B/5A	−0.003621166767606
u_{23}	2A/5A	−0.001817127650116	u_{47}	4B/6A	−0.005318237146257
u_{24}	2B/2B	0.000000000000000	u_{48}	5A/5A	−0.004859306805426

General Energy Model for PAHs from C_6H_6 to $C_{96}H_{24}$

For the general model for predicting reference reaction energies (ΔE_{ref}, in kcal/mol), trained on the 2,275 reference PAH molecules ranging from C_6H_6 to $C_{96}H_{24}$, the optimized parameters (Eq. 5 in the main text) are:

- $\beta = -56.303406215855262$ kcal/mol
- $\gamma = -71.0805880111976$ kcal/mol, $\lambda = -0.3496263686$, $d_0 = 1.2067223668$ Å
- $\eta = 1.383132975297873$ kcal/mol
- $\nu = 281.936940193539385$ kcal/mol
- $\mu = -72.049728013506765$ kcal/mol
- $\epsilon = 4.371419145837001$ kcal/mol

Bond Length Model for PAHs from C_6H_6 to $C_{96}H_{24}$

For the bond length model applied to PAHs ranging from C_6H_6 to $C_{96}H_{24}$, we adopted the optimized hyperparameter $\zeta = 2.70$ from the model for $C_{48}H_{24}$ PAHs. We then performed a least-squares fit (Eq. 3 in the main text) to the DFT-optimized C−C bond lengths across all 2,275 reference PAHs. The optimized parameters are listed below and in Tables S3 and S4.

- $\zeta = 2.70$
- $\rho = 0.144462739934206$

Table S3. Optimized values (in Å) of parameters for different atom-based types of C−C bonds in the bond length model for PAHs from C_6H_6 to $C_{96}H_{24}$. See Section for definitions of atom-based bond types.

Parameter	Bond type	Value	Parameter	Bond type	Value
w_1	Type 1	1.319254373298919	w_8	Type 8	1.344060028378755
w_2	Type 2	1.317978265412778	w_9	Type 9	1.343143159231143
w_3	Type 3	1.317334834379969	w_{10}	Type 10	1.332864799546223
w_4	Type 4	1.315804760245559	w_{11}	Type 11	1.329361425967363
w_5	Type 5	1.322646494018396	w_{12}	Type 12	1.338800895300115
w_6	Type 6	1.318365595258982	w_{13}	Type 13	1.338021479421883
w_7	Type 7	1.322972489501888	w_{14}	Type 14	1.335011223454159

Table S4. Optimized values (in Å) of parameters for different ring-based types of C−C bonds in the bond length model for PAHs from C_6H_6 to $C_{96}H_{24}$. See Section for definitions of ring-based bond types.

Parameter	Bond type	Value	Parameter	Bond type	Value
u_1	1A	0.015675270192122	u_{28}	2C/2C	−0.008360443134440
u_2	2A	0.013894823925780	u_{29}	2C/3B	−0.005326591530247
u_3	2B	0.010050003828004	u_{30}	2C/3C	0.012434108645148
u_4	2C	0.011673466560773	u_{31}	2C/4B	0.011560649007106
u_5	3A	0.011306179626505	u_{32}	3A/3A	0.002886349201679
u_6	3B	0.006794235506993	u_{33}	3A/3B	0.003681062130366
u_7	3C	0.003048290574175	u_{34}	3A/4A	0.000855143981080
u_8	4A	0.008278261799653	u_{35}	3A/4B	0.001184346778623
u_9	4B	0.002276858296238	u_{36}	3A/4C	0.000496897415995
u_{10}	4C	0.002158950548404	u_{37}	3A/5A	0.000371538802602
u_{11}	5A	0.001994508920933	u_{38}	3A/6A	−0.001982048585742
u_{12}	1A/1A	0.000000000000000	u_{39}	3B/3B	−0.001230041804702
u_{13}	1A/2B	0.000000000000000	u_{40}	3B/4A	0.004319202108558
u_{14}	1A/2C	−0.005217527029099	u_{41}	3B/4B	−0.000739836549236
u_{15}	1A/3B	−0.004213847708764	u_{42}	3B/4C	−0.002038241499547
u_{16}	1A/3C	0.013932737345404	u_{43}	3B/5A	−0.002269418075408
u_{17}	1A/4B	0.012537594934331	u_{44}	4A/4A	−0.001106039706674
u_{18}	2A/2A	0.010702527806280	u_{45}	4A/4B	−0.000238845912824
u_{19}	2A/3A	0.006651034845353	u_{46}	4A/4C	−0.001792887883440
u_{20}	2A/3B	0.005074682053504	u_{47}	4A/5A	−0.000559896241102
u_{21}	2A/4A	0.003928722603235	u_{48}	4A/6A	−0.001988869639665
u_{22}	2A/4B	0.003804785254527	u_{49}	4B/4B	0.000000000000000
u_{23}	2A/4C	0.002148600632742	u_{50}	4B/5A	−0.000723997909945
u_{24}	2A/5A	0.000671047628549	u_{51}	4B/6A	−0.002949967859207
u_{25}	2B/2B	0.005344652755440	u_{52}	5A/5A	−0.000417516976095
u_{26}	2B/2C	−0.002399980633527	u_{53}	5A/6A	−0.001838688488872
u_{27}	2B/3B	0.004504409885767	u_{54}	6A/6A	−0.002616489721736

Note 10: Bond Types for Predicting Bond Lengths

In PAHs composed of fully fused rings (i.e., without any terminal carbon atoms outside the rings), all carbon atoms are trivalent (sp^2 hybridized) and are classified as either "C" (bonded to three carbon atoms) or "CH" (bonded to two carbon atoms and one hydrogen atom). As a result, three types of C−C bonds are possible: CH−CH, CH−C, and C−C bonds, corresponding to the central bonds highlighted in black in Fig. S8. By further distinguishing these bonds according to the types of their neighboring carbon atoms (shown in blue), we identify 14 distinct atom-based C−C bond types, as summarized in Fig. S8.

A more refined classification of C−C bonds in fully fused PAHs can be achieved by considering the types of the two rings that share a given bond. To this end, we categorize individual rings into twelve types based on both the number and spatial arrangement of their neighboring rings, as illustrated in Fig. S9. Each ring type is labeled with a number indicating the count of neighboring rings, followed by a letter (A, B, or C) to distinguish different arrangements of these neighboring rings. The ring-based type of a given C−C bond is then defined as follows: if the bond belongs exclusively to one ring, its type is simply that of the ring itself (e.g., 1A, 2B, or 3C); if it is shared by two rings, then its type is defined by the types of those two rings, and is labeled accordingly using the pair of ring type labels (e.g., 1A/2C, 3B/3B, or 4C/5A).

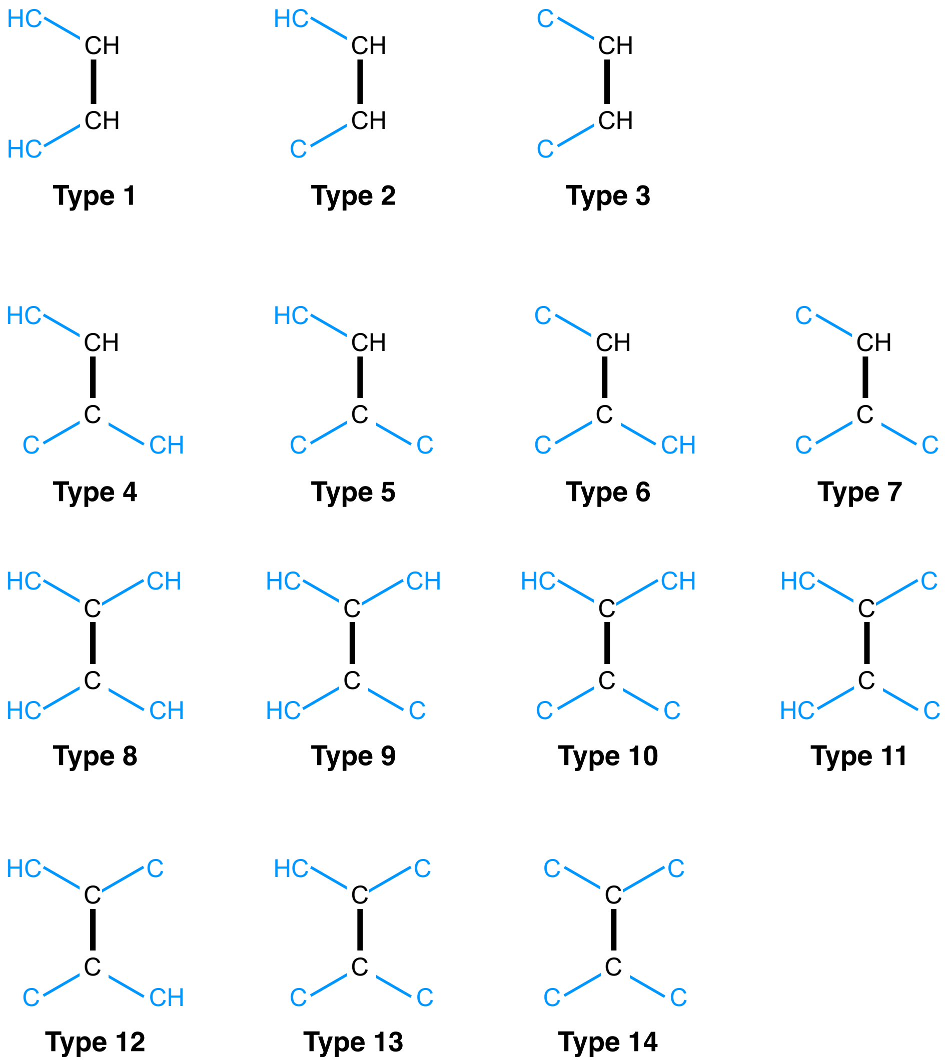

Figure S8. Fourteen atom-based types of C−C bonds found in fully fused PAHs, classified according to the bonding environments of the two carbon atoms forming the bond (indicated by bold black lines) and those of their neighboring carbon atoms (in blue). The bonding environment refers to the number of hydrogen atoms attached to each carbon atom, being either one hydrogen (CH) or none (C).

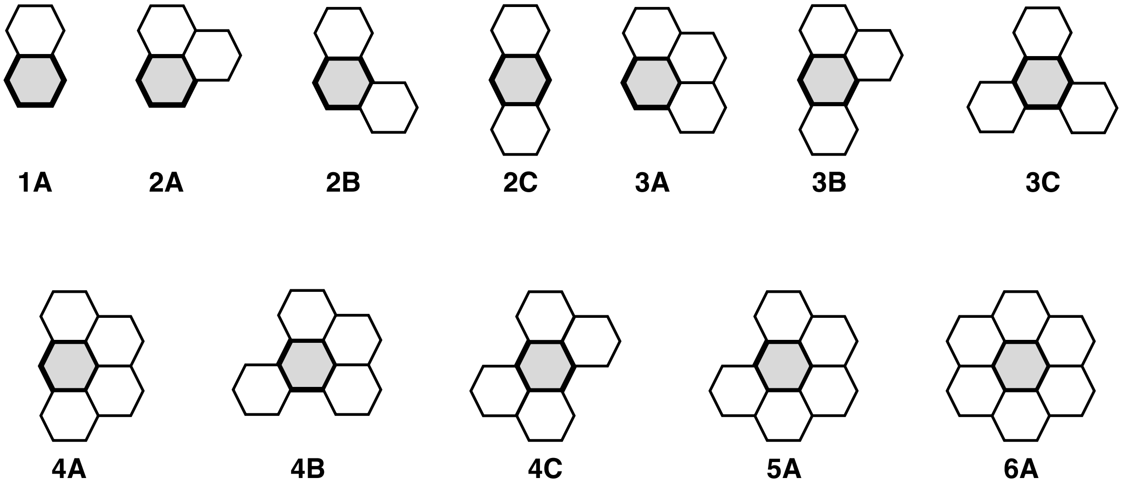

Figure S9. Classification of rings in PAHs based on the number and arrangement of neighboring rings (unshaded) surrounding a given ring (shaded).

Note 11: Validation of Bond Length Models on Benchmark CNBs

To further evaluate the generalization ability of our bond length models (see Eq. 7 in the main text), we test their performance in predicting equilibrium C−C bond lengths for ten large CNBs, each comprising 120 rings. These [120]CNBs contain 10 times as many the repeat units as the ten benchmark [12]CNBs used in the main text (see Fig. 3a for their structures). With a total of 120 rings, each CNB is sufficiently large to approximate the corresponding strainless reference CNB in the infinite-size limit. Importantly, none of these [120]CNBs were included in the training sets for our bond length models.

As shown in Fig. S10, both models (one trained on the 90,960 C−C bonds in the 1,516 reference $C_{48}H_{24}$ PAH molecules, and the other on the 205,350 bonds in the 2,275 reference PAHs from C_6H_6 to $C_{96}H_{24}$) show strong predictive performance, with $R^2 \approx 0.995$ and RMSE ≈ 0.002 Å.

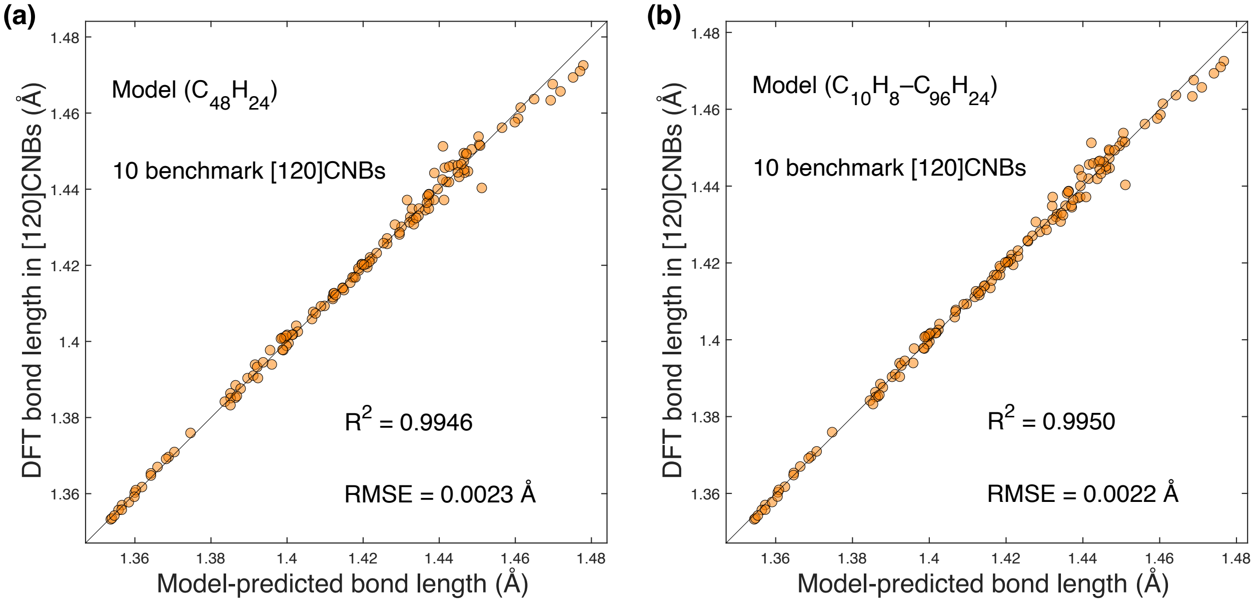

Figure S10. Comparison between model-predicted equilibrium C−C bond lengths and those obtained from DFT geometry optimizations for ten benchmark CNBs containing 120 rings. All C−C bond lengths in the repeat unit of these CNBs are included. (a) Predictions from the model trained on the 90,960 C−C bonds in the 1,516 reference $C_{48}H_{24}$ PAHs. (b) Predictions from the model trained on the 205,350 C−C bonds in the 2,275 reference PAHs ranging from C_6H_6 to $C_{96}H_{24}$.

Note 12: Performance of Models Employing the Simple and Distance-Dependent HMO Methods

Figure S11 shows that incorporating the distance-dependent HMO method into the energy prediction model provides negligible improvement in performance compared with the model based on the simple HMO theory.

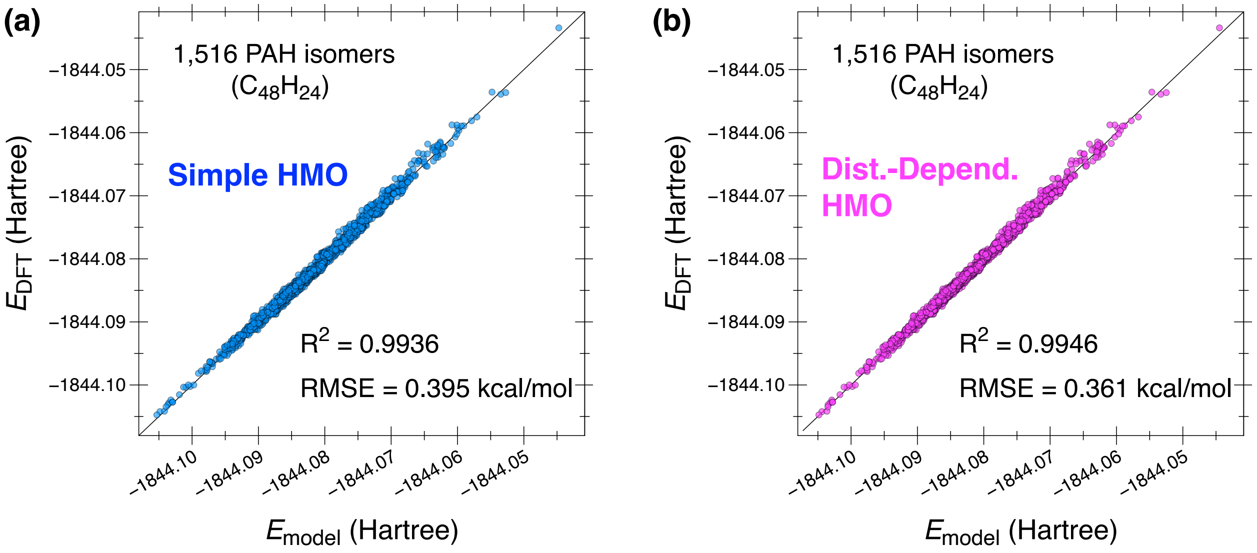

Figure S11. Comparison of model performance in predicting DFT total energies of $C_{48}H_{24}$ benzenoid PAHs using (a) the simple HMO theory and (b) the distance-dependent HMO method.

Figure S12 shows that the simple HMO theory performs similarly, though slightly less accurately, than the distance-dependent HMO method in predicting DFT-optimized C−C bond lengths for planar benzenoid PAHs of various sizes. However, the SEs predicted using the simple HMO model are significantly underestimated for the Vögtle belt and its Möbius isomer (62.1 and 192.6 kcal/mol, respectively) compared with the values obtained from StrainViz calculations (105 and 238 kcal/mol);[3] see Fig. S13 for the molecular structures of Vögtle belt and its Möbius form. In contrast, the models using the distance-dependent HMO method yields SE predictions (101.6 and 232.1 kcal/mol) in close agreement with the StrainViz results. The reason for the failure of the simple HMO method in these cases is that the Coulson bond orders from the simple HMO framework correlate poorly with the bond lengths (with R^2 of only ca. 0.5), as shown in Figs. S14a and S15a. In comparison, the distance-dependent HMO method produces Coulson bond orders that correlate strongly with bond lengths ($R^2 > 0.92$), as shown in Figs. S14b and S15b.

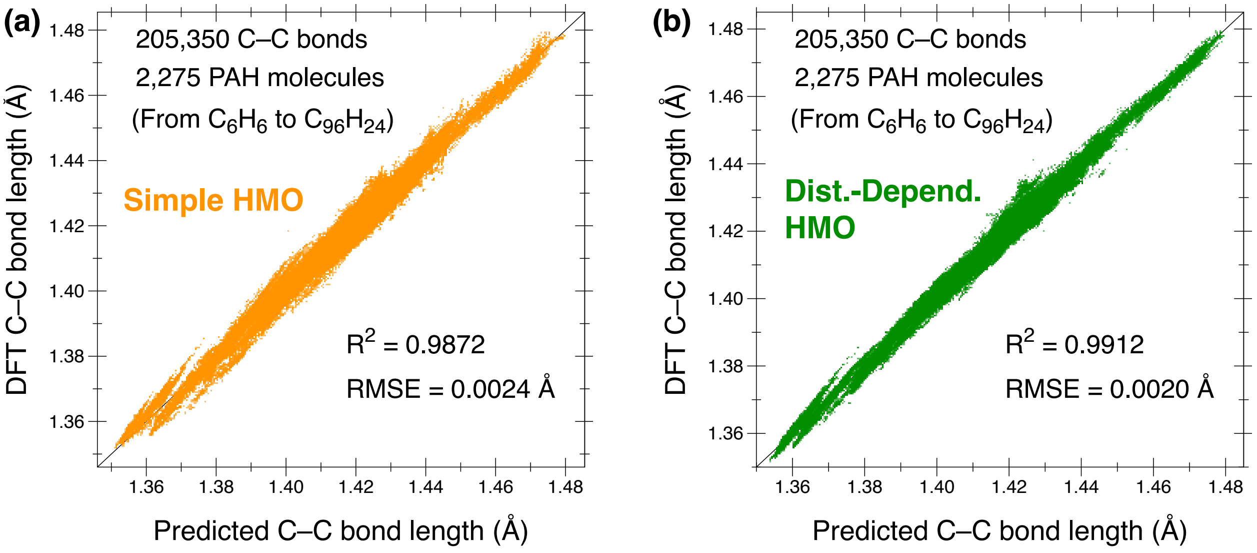

Figure S12. Comparison of model performance in predicting DFT-optimized C–C bond lengths for benzenoid PAHs ranging from C_6H_6 to $C_{96}H_{24}$ using (a) the simple HMO theory and (b) the distance-dependent HMO method.

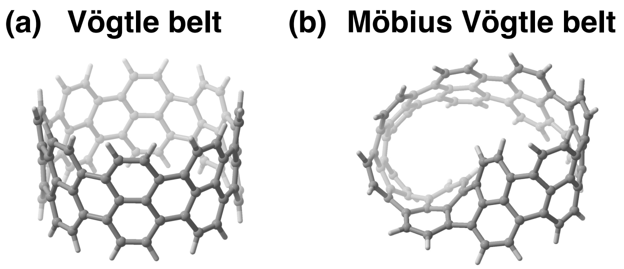

Figure S13. Structures of (a) the Vögtle belt[37] and (b) its Möbius form.[3]

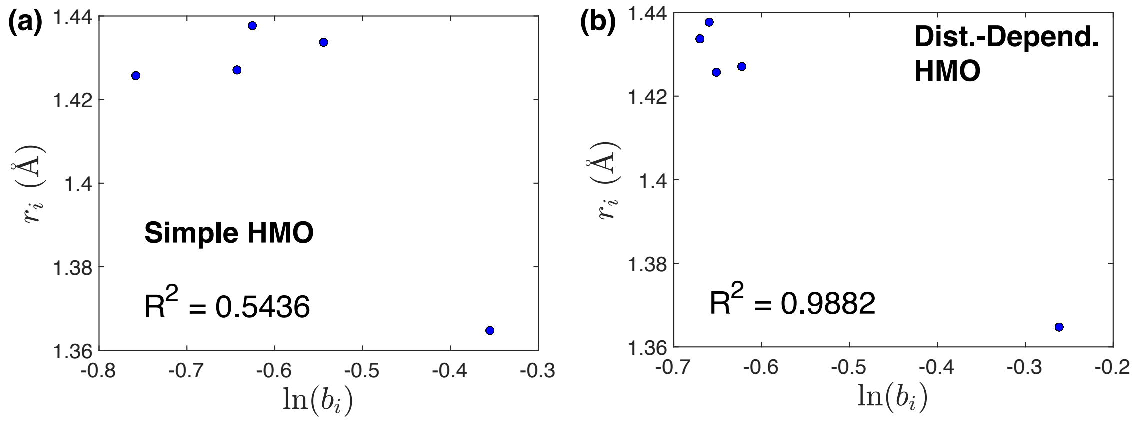

Figure S14. DFT-optimized C−C bond lengths (d_i) of the Vögtle belt plotted against the logarithm of the corresponding Coulson bond orders ($\ln(b_i)$) calculated using (a) the simple HMO method and (b) the distance-dependent HMO method.

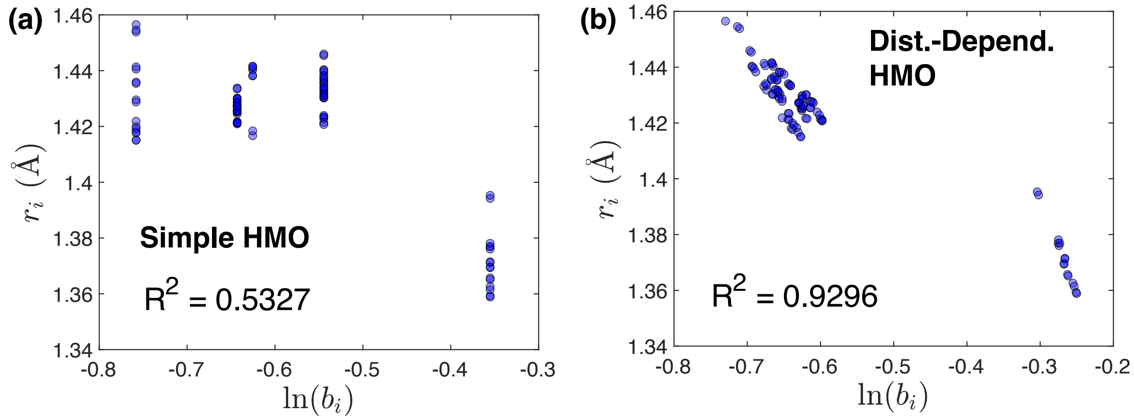

Figure S15. Same as Fig. S14, but for the Möbius form of the Vögtle belt.

Note 13: Validation of Ground-State Diagnosis by UBS-DFT

Given the large number (> 2,000) and broad size range (up to $C_{96}H_{24}$) of molecules in our dataset, it is impractical to perform the computationally very demanding multiconfigurational ab initio calculations. Therefore, we employed the UBS-DFT approach at the B3LYP/6-311G* level for all generated PAHs for open-shell calculations. This method has been widely applied to diradical and polyradical PAHs.[38–44] It has been shown to yield reasonable results, supported by high-level multiconfigurational methods such as CASSCF and MR-MP2.[32,45,46] In particular, hybrid functionals (such as B3LYP employed in this work) are shown to outperform pure DFT functionals, as they mitigate self-interaction error and better capture static electron correlation.[47]

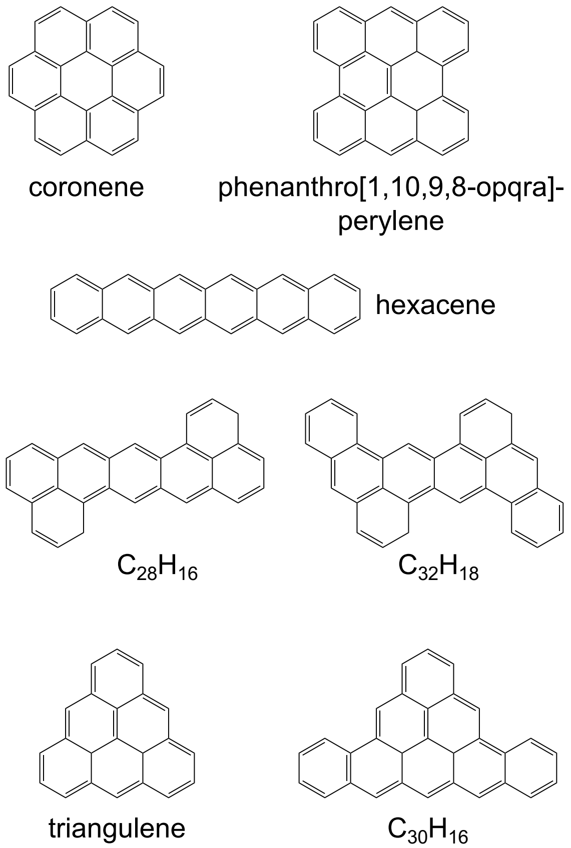

Figure S16. Chemical structures of test PAHs for CASSCF and NEVPT2 calculations.

To further validate our UBS-DFT results, we selected several representative PAHs of moderate size (for the sake of computational feasibility) and performed additional CASSCF and NEVPT2 single-point calculations using the def2-SVP basis set using the ORCA program.[48] The chemical structures of these test PAHs are shown in Fig. S16. Specifically, state-averaged (SA) CASSCF(10,10) calculations were carried out over two singlet and two

triplet roots. Our choice of active space sizes appears reasonable since in a recent study[49] CAS(8,8) reference spaces were used to compute the lowest singlet/triplet states of comparably sized PAHs exhibiting polyradical character.

Table S5. Comparison of SA-CASSCF(10,10), NEVPT2, and UBS-DFT results for representative PAHs in the dataset.

Molecule	Ground state	ΔE(T–S)^a (kcal/mol)			c_1, c_2 (CASSCF)^b
		CASSCF	NEVPT2	UBS-DFT	
coronene	Closed-shell	81.8	74.2	67.1	0.88094, 0.01411
$C_{28}H_{14}{}^c$	Closed-shell	32.4	23.4	18.8	0.86421, 0.03362
hexacene	Open-shell singlet	18.6	18.0	12.8	0.62117, 0.12692
$C_{28}H_{16}{}^d$	Open-shell singlet	24.9	20.2	12.1	0.76541, 0.07626
$C_{32}H_{18}{}^d$	Open-shell singlet	11.0	12.3	7.1	0.68402, 0.16192
triangulene	Triplet	−20.9	−12.5	−7.1	—
$C_{30}H_{16}{}^d$	Triplet	−20.3	−12.0	−5.6	—

^{a}Energy difference between the lowest triplet and singlet states, defined as E(triplet) − E(singlet).
^{b}Configuration interaction (CI) coefficients for the two leading configuration state functions (CSFs) in the ground-state CASSCF wave function.
^{c}Phenanthro[1,10,9,8-opqra]perylene; see Fig. S16 for its chemical structure.
^{d}See Fig. S16 for their chemical structures.

As shown in Table S5, the UBS-DFT approach provides a qualitatively reliable ground-state diagnosis for the systems studied. The singlet–triplet energy gaps, ΔE(T–S), predicted by UBS-DFT are of the same order of magnitude as those obtained from NEVPT2 calculations. For coronene and phenanthro[1,10,9,8-opqra]perylene ($C_{28}H_{14}$), the CI coefficient of the leading CSF (> 0.86) is substantially larger than those of minor CSFs (< 0.04), indicating that these molecules possess closed-shell ground states. In contrast, for hexacene, $C_{28}H_{16}$, and $C_{32}H_{18}$, the leading CI coefficient decreases significantly (0.62–0.76), suggesting pronounced diradical/polyradical character. These results are consistent with the UBS-DFT ground-state diagnosis.

Supplementary References

(1) Hansen, P.; Lebatteux, C.; Zheng, M. The boundary-edges code for polyhexes. *J. Mol. Struct. (Theochem)* **1996**, *363*, 237–247.

(2) Herndon, W.; Bruce, A. Perimeter code for benzenoid aromatic hydrocarbons. *Stud. Phys. Theor. Chem.* **1987**, *51*, 491–513.

(3) Colwell, C. E.; Price, T. W.; Stauch, T.; Jasti, R. Strain visualization for strained macrocycles. *Chem. Sci.* **2020**, *11*, 3923–3930.

(4) Diederich, F.; Staab, H. A. Benzenoid versus Annulenoid Aromaticity: Synthesis and Properties of Kekulene. *Angew. Chem. Int. Ed.* **1978**, *17*, 372–374.

(5) Krieger, C.; Diederich, F.; Schweitzer, D.; Staab, H. A. Molecular Structure and Spectroscopic Properties of Kekulene. *Angew. Chem. Int. Ed.* **1979**, *18*, 699–701.

(6) Staab, H. A.; Diederich, F. Cycloarenes, a New Class of Aromatic Compounds, I. Synthesis of Kekulene. *Chem. Ber.* **1983**, *116*, 3487–3503.

(7) Staab, H. A.; Diederich, F.; Krieger, C.; Schweitzer, D. Cycloarenes, a New Class of Aromatic Compounds, II. Molecular Structure and Spectroscopic Properties of Kekulene. *Chem. Ber.* **1983**, *116*, 3504–3512.

(8) Pozo, I. et al. Revisiting Kekulene: Synthesis and Single-Molecule Imaging. *J. Am. Chem. Soc.* **2019**, *141*, 15488–15493.

(9) Cheung, K. Y.; Segawa, Y.; Itami, K. Synthetic Strategies of Carbon Nanobelts and Related Belt-Shaped Polycyclic Aromatic Hydrocarbons. *Chem. Eur. J.* **2020**, *26*, 14791–14801.

(10) Guo, Q.-H.; Qiu, Y.; Wang, M.-X.; Stoddart, J. F. Aromatic hydrocarbon belts. *Nat. Chem.* **2021**, *13*, 402–419.

(11) Li, Y.; Kono, H.; Maekawa, T.; Segawa, Y.; Yagi, A.; Itami, K. Chemical Synthesis of Carbon Nanorings and Nanobelts. *Acc. Mater. Res.* **2021**, *2*, 681–691.

(12) Imoto, D.; Yagi, A.; Itami, K. Carbon Nanobelts: Brief History and Perspective. *Precis. Chem.* **2023**, *1*, 516–523.

(13) Zhang, R.; An, D.; Zhu, J.; Lu, X.; Liu, Y. Carbon Nanorings and Nanobelts: Material Syntheses, Molecular Architectures, and Applications. *Adv. Funct. Mater.* **2023**, *33*, 2305249.

(14) Segawa, Y.; Yagi, A.; Ito, H.; Itami, K. A Theoretical Study on the Strain Energy of Carbon Nanobelts. *Org. Lett.* **2016**, *18*, 1430–1433.

(15) Povie, G.; Segawa, Y.; Nishihara, T.; Miyauchi, Y.; Itami, K. Synthesis of a carbon nanobelt. *Science* **2017**, *356*, 172–175.

(16) Povie, G.; Segawa, Y.; Nishihara, T.; Miyauchi, Y.; Itami, K. Synthesis and Size-Dependent Properties of [12], [16], and [24]Carbon Nanobelts. *J. Am. Chem. Soc.* **2018**, *140*, 10054–10059.

(17) Bergman, H. M.; Kiel, G. R.; Handford, R. C.; Liu, Y.; Tilley, T. D. Scalable, Divergent Synthesis of a High Aspect Ratio Carbon Nanobelt. *J. Am. Chem. Soc.* **2021**, *143*, 8619–8624.

(18) Segawa, Y. et al. Synthesis of a Möbius carbon nanobelt. *Nat. Synth.* **2022**, *1*, 535–541.

(19) Tibbetts, G. G. Why are carbon filaments tubular? *J. Cryst. Growth* **1984**, *66*, 632–638.

(20) Robertson, D. H.; Brenner, D. W.; Mintmire, J. W. Energetics of nanoscale graphitic tubules. *Phys. Rev. B* **1992**, *45*, 12592–12595.

(21) Kumar, B. et al. Septulene: The Heptagonal Homologue of Kekulene. *Angew. Chem. Int. Ed.* **2012**, *51*, 12795–12800.

(22) Biedermann, P. U.; Pogodin, S.; Agranat, I. Inversion Barrier of Corannulene. A Benchmark for Bowl-to-Bowl Inversions in Fullerene Fragments. *J. Org. Chem.* **1999**, *64*, 3655–3662.

(23) Ehrenhauser, F. S. PAH and IUPAC Nomenclature. *Polycycl. Aromat. Comp.* **2015**, *35*, 161–176.

(24) Ruiz-Morales, Y.; Alvarez-Ramírez, F. Usage of the Y-Rule and the Effect of the Occurrence of Heteroatoms (N, S) on the Frontier Molecular Orbitals Gap of Polycyclic Aromatic Hydrocarbons (PAHs), and Asphaltene-PAHs. *ChemPhysChem* **2023**, *24*, e202200682.

(25) Caporossi, G.; Hansen, P. Enumeration of Polyhex Hydrocarbons to $h = 21$. *J. Chem. Inf. Comput. Sci.* **1998**, *38*, 610–619.

(26) Hückel, E. Quantentheoretische Beiträge zum Benzolproblem. I. Die Elektronenkonfiguration des Benzols und verwandter Verbindungen. *Z. Phys.* **1931**, *70*, 204–286.

(27) Hückel, E. Quantentheoretische Beiträge zum Benzolproblem. II. Quantentheorie der induzierten Polaritäten. *Z. Phys.* **1931**, *72*, 310–337.

(28) Hückel, E. Quantentheoretische Beiträge zum Problem der aromatischen und ungesättigten Verbindungen. III. *Z. Phys.* **1932**, *72*, 628–648.

(29) Hückel, E. Die freien Radikale der organischen Chemie. Quantentheoretische Beiträge zum Problem der aromatischen und ungesättigten Verbindungen. IV. *Z. Phys.* **1933**, *83*, 632–668.

(30) Grimme, S.; Bannwarth, C.; Shushkov, P. A Robust and Accurate Tight-Binding Quantum Chemical Method for Structures, Vibrational Frequencies, and Noncovalent Interactions of Large Molecular Systems Parametrized for All spd-Block Elements (Z = 1–86). *J. Chem. Theory Comput.* **2017**, *13*, 1989–2009.

(31) Bannwarth, C.; Ehlert, S.; Grimme, S. GFN2-xTB—An Accurate and Broadly Parametrized Self-Consistent Tight-Binding Quantum Chemical Method with Multipole Electrostatics and Density-Dependent Dispersion Contributions. *J. Chem. Theory Comput.* **2019**, *15*, 1652–1671.

(32) Chen, Z. et al. Open-Shell Singlet Character of Cyclacenes and Short Zigzag Nanotubes. *Org. Lett.* **2007**, *9*, 5449–5452.

(33) Oiwa, M.; Ryoshikagaku, S. *Elementary Quantum Chemistry*; Kagaku-Dojin: Kyoto, 1965.

(34) Sato, T.; Tanaka, M.; Yamabe, T. Size-dependent HOMO-LUMO gap oscillation of carbon nanotube with a finite length. *Synth. Met.* **1999**, *103*, 2525–2526.

(35) Poater, J.; Visser, R.; Solà, M.; Bickelhaupt, F. M. Polycyclic Benzenoids: Why Kinked is More Stable than Straight. *J. Org. Chem.* **2007**, *72*, 1134–1142.

(36) Poater, J.; Duran, M.; Solà, M. Aromaticity Determines the Relative Stability of Kinked vs. Straight Topologies in Polycyclic Aromatic Hydrocarbons. *Front. Chem.* **2018**, *6*, 561.

(37) Vögtle, F.; Schröder, A.; Karbach, D. Strategy for the Synthesis of Tube-Shaped Molecules. *Angew. Chem. Int. Ed. Engl.* **1991**, *30*, 575–577.

(38) Jiang, D.-e.; Dai, S. Electronic Ground State of Higher Acenes. *J. Phys. Chem. A* **2008**, *112*, 332–335.

(39) Motomura, S. et al. Size dependences of the diradical character and the second hyperpolarizabilities in dicyclopenta-fused acenes: relationships with their aromaticity/antiaromaticity. *Phys. Chem. Chem. Phys.* **2011**, *13*, 20575–20583.

(40) Morita, Y.; Suzuki, S.; Sato, K.; Takui, T. Synthetic organic spin chemistry for structurally well-defined open-shell graphene fragments. *Nat. Chem.* **2011**, *3*, 197–204.

(41) Huang, B.; Kang, H.; Zhang, C.-W.; Zhao, X.-L.; Shi, X.; Yang, H.-B. Design of an open-shell nitrogen-centered diradicaloid with tunable stimuli-responsive electronic properties. *Commun. Chem.* **2022**, *5*, 127.

(42) Borissov, A.; Chmielewski, P. J.; Gómez García, C. J.; Lis, T.; Stępień, M. Dinor[7]helicene and Beyond: Divergent Synthesis of Chiral Diradicaloids with Variable Open-Shell Character. *Angew. Chem. Int. Ed.* **2023**, *62*, e202309238.

(43) Weng, T. et al. 1,1′-Biolympicenyl: A Stable Non-Kekulé Diradical with a Small Singlet and Triplet Energy Gap. *J. Am. Chem. Soc.* **2024**, *146*, 26454–26465.

(44) He, C.-C.; Zeng, J.; Zhao, Y.-J.; Yang, X.-B. Unified bonding entropy model to determine magnetic properties in graphene nanoflakes. *Phys. Rev. B* **2025**, *112*, 094404.

(45) Bendikov, M.; Duong, H. M.; Starkey, K.; Houk, K. N.; Carter, E. A.; Wudl, F. Oligoacenes: Theoretical Prediction of Open-Shell Singlet Diradical Ground States. *J. Am. Chem. Soc.* **2004**, *126*, 7416–7417.

(46) Marković, S.; Đurđević, J.; Jeremić, S.; Gutman, I. Triplet fluoranthenes: Aromaticity versus unpaired electrons. *J. Mol. Model.* **2011**, *17*, 805–810.

(47) Gräfenstein, J.; Kraka, E.; Filatov, M.; Cremer, D. Can Unrestricted Density-Functional Theory Describe Open Shell Singlet Biradicals? *Int. J. Mol. Sci.* **2002**, *3*, 360–394.

(48) Neese, F. The ORCA program system. *WIRES Comput. Molec. Sci.* **2012**, *2*, 73–78.

(49) Nieman, R. et al. Polyradical character assessment using multireference calculations and comparison with density-functional derived fractional occupation number weighted density analysis. *Phys. Chem. Chem. Phys.* **2023**, *25*, 27380–27393.
