# Supplementary material for: Autodesmotic reactions for general strain energy evaluation in polycyclic aromatic nanocarbons
Source: Commun Chem. 2025 Dec 16;9:41. doi: 10.1038/s42004-025-01848-w (PMC12824410; doi:10.1038/s42004-025-01848-w)
Supplement: Supplementary file 3 — Description of Additional Supplementary Files [file 42004_2025_1848_MOESM3_ESM.pdf]

Description of Additional Supplementary Files:

File: Supplementary Data 1

Description: .zip archive containing .xyz files with Cartesian coordinates and absolute energies of all molecules used for SE evaluation.

File: Supplementary Data 2

Description: Source data underlying Figure 3.

File: Supplementary Data 3

Description: Source data underlying Figure 4.

File: Supplementary Data 4

Description: Source data underlying Figure 6.

File: Supplementary Data 5

Description: Source data underlying Figure 7.

File: Supplementary Data 6

Description: Source data underlying Figure 8.
